# Ultrasound genomics related mitochondrial gene signature for prognosis and neoadjuvant chemotherapy resistance in triple negative breast cancer

**DOI:** 10.32604/or.2024.054642

**Published:** 2025-02-28

**Authors:** HUAFANG HUANG, GUILIN WANG, DONGYUN ZENG, LUZ ANGELA TORRES-DE LA ROCHE, RUI ZHUO, RUDY LEON DE WILDE, WANWAN WANG, ULF D. KAHLERT, WENJIE SHI

**Affiliations:** 1 Department of Breast Surgery, EUSOMA Certificate Breast Cancer Center (No.1037/00), Guilin TCM Hospital of China, Guilin, 541002, China; 2 University Hospital for Gynecology, Pius-Hospital, University Medicine Oldenburg, Oldenburg, 26121, Germany; 3 Department of Breast and Thyroid Surgery, The Second Affiliated Hospital of Guilin Medical University, Guilin, 541002, China; 4 Clinicopathological Diagnosis & Research Center, The Affiliated Hospital of Youjiang Medical University for Nationalities, Baise, 533000, China; 5 Key Laboratory of Tumor Molecular Pathology of Guangxi Higher Education Institutes, Baise, 533000, China; 6 Department of Breast and Thyroid Surgery, Xuzhou No.1 People’s Hospital, Xuzhou, 221000, China; 7 Molecular and Experimental Surgery, University Clinic for General-, Visceral-, Vascular- and Trans-Plantation Surgery, Medical Faculty University Hospital Magdeburg, Otto-von Guericke University, Magdeburg, 39120, Germany

**Keywords:** Ultrasound genomics, Mitochondrial, Breast cancer, Neoadjuvant, Resistance

## Abstract

**Background:**

Neoadjuvant chemotherapy (NAC) significantly enhances clinical outcomes in patients with triple-negative breast cancer (TNBC); however, chemoresistance frequently results in treatment failure. Consequently, understanding the mechanisms underlying resistance and accurately predicting this phenomenon are crucial for improving treatment efficacy.

**Methods:**

Ultrasound images from 62 patients, taken before and after neoadjuvant therapy, were collected. Mitochondrial-related genes were extracted from a public database. Ultrasound features associated with NAC resistance were identified and correlated with significant mitochondrial-related genes. Subsequently, a prognostic model was developed and evaluated using the GSE58812 dataset. We also assessed this model alongside clinical factors and its ability to predict immunotherapy response.

**Results:**

A total of 32 significant differentially expressed genes in TNBC across three groups indicated a strong correlation with ultrasound features. Univariate and multivariate Cox regression analyses identified six genes as independent risk factors for TNBC prognosis. Based on these six mitochondrial-related genes, we constructed a TNBC prognostic model. The model’s risk scores indicated that high-risk patients generally have a poorer prognosis compared to low-risk patients, with the model demonstrating high predictive performance (*p* = 0.002, AUC = 0.745). This conclusion was further supported in the test set (*p* = 0.026, AUC = 0.718). Additionally, we found that high-risk patients exhibited more advanced tumor characteristics, while low-risk patients were more sensitive to common chemotherapy drugs and immunotherapy. The signature-related genes also predicted immunotherapy response with a high accuracy of 0.765.

**Conclusion:**

We identified resistance-related features from ultrasound images and integrated them with genomic data, enabling effective risk stratification of patients and prediction of the efficacy of neoadjuvant chemotherapy and immunotherapy in patients with TNBC.

## Introduction

Breast cancer remains the second leading cause of cancer-related deaths among women, with incidence rates rising significantly over the past four decades. In 2020, approximately 2.3 million new cases were reported globally, resulting in around 685,000 deaths, with prevalence and mortality rates varying widely across different economic regions [1]. Clinically, breast tumors are categorized into three primary groups based on the presence of estrogen receptor (ER), progesterone receptor (PR), and human epidermal growth factor receptor 2 (HER2): ER-positive (ER+), HER2-positive (HER2+), and triple-negative breast cancer (TNBC) [2]. TNBC, which accounts for about 15% of breast cancer cases, primarily affects younger women and is often diagnosed at a higher grade, contributing disproportionately to breast cancer mortality [2,3]. Due to the absence of ER, PR, and HER2 receptors, treatments effective for other breast cancer subtypes are not suitable for TNBC. The molecular diversity, varied pathology, and distinct clinical features of TNBC result in different sensitivities to various chemotherapy drugs [3,4]. Given the heterogeneous biological characteristics and aggressive clinical progression of TNBC, molecular profiling through the identification of gene expression is crucial for risk stratification and precision treatment of this condition.

Over the past decade, genomics-based molecular profiling studies have significantly advanced and been successfully implemented in clinical practice, benefiting patients with TNBC [5]. For instance, gene expression analysis has identified two distinct subtypes of TNBC, each exhibiting unique responses to various therapies. These subtypes include two oxidative stress-related groups, each characterized by specific gene signatures. The study underscored the effectiveness of immune checkpoint inhibitors (ICIs) for TNBC subtypes, supporting personalized treatment approaches [6]. Researchers from Fudan University proposed the Fudan subtypes for TNBC based on mRNA expression, identifying four distinct subtypes with different prognoses and suggesting specific targeted therapies for each. This demonstrates the potential for more effective, personalized treatments for TNBC [7]. These studies illuminate new avenues for treating and predicting the prognosis of TNBC and emphasize the potential for personalized therapies tailored to distinct molecular subtypes.

Mitochondria, as the only extranuclear organelles containing genetic material, play a crucial role in carcinogenesis through their communication with and retrograde regulation of the nucleus [8]. Reactive oxygen species (ROS) within mitochondria have been implicated in promoting tumor cell proliferation, migration, and apoptosis [9]. Research has shown that TNBC cells exhibit distinct metabolic alterations, such as high glucose uptake, increased lactate production, and reduced mitochondrial respiration, compared to other breast cancer subtypes. This suggests that TNBC cells may be particularly susceptible to treatments targeting glycolytic pathways, presenting a potentially effective therapeutic strategy for this aggressive cancer subtype [10]. Additionally, recent advancements emphasize the role of mitochondria in the tumor microenvironment (TME), contributing to the aggressiveness of TNBC [11,12]. One study found that targeting the prooxidant effects of manganese superoxide dismutase can significantly inhibit TNBC progression and reduce the functionality of M2 macrophages, which typically support tumor growth under oncogenic stress. By reducing mitochondrial ROS, this approach can mitigate the immunosuppressive TME and curb the advancement of TNBC [12].

Building on the aforementioned research, neoadjuvant therapy has emerged as a key strategy for treating TNBC, significantly reducing tumor burden in patients. However, current methods for assessing therapeutic response primarily rely on clinical and pathological indicators, which have limitations, such as subjective evaluation criteria and the inability to fully and accurately predict treatment outcomes. The development of ultrasound genomics, powered by deep learning technology, offers a promising solution to these challenges. Recent breakthroughs in medical-industrial cross-research have advanced ultrasound genomics in predicting tumor treatment efficacy. In particular, ultrasonic genomics enables precise localization and classification of key targets from visual data, such as images and videos, providing valuable guidance for early tumor signal recognition.

In this study, we aimed to identify resistance-related features and classify TNBC using ultrasound genomics. By analyzing risk stratification, we will examine the chemotherapy sensitivity and mitochondria-related gene expression profiles of each group to develop personalized treatment strategies. This approach has the potential to offer an effective therapeutic strategy for this aggressive breast cancer subtype.

## Materials and Methods

### Data acquisition and preprocessing

**Figure 1:**
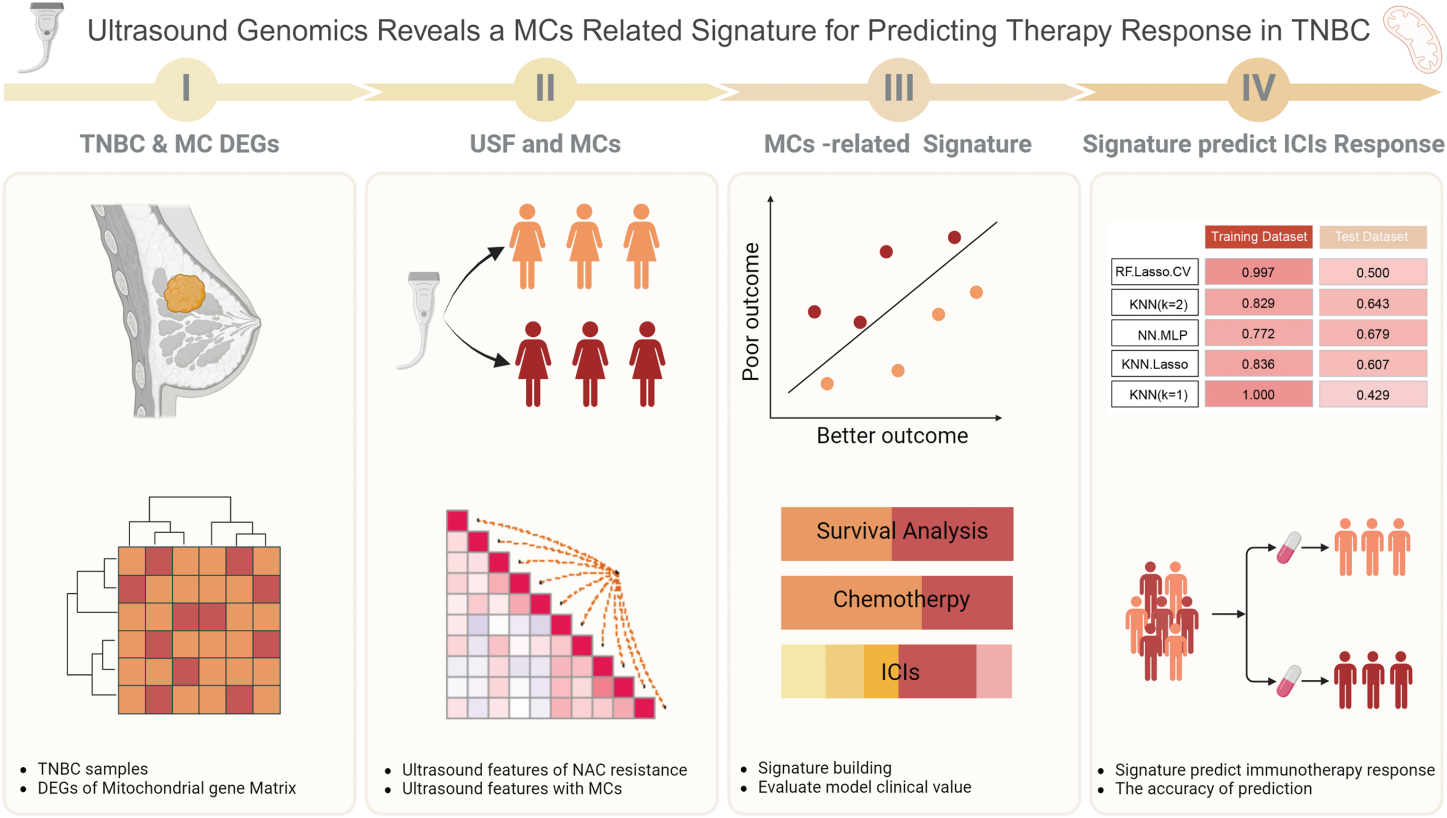
Workflow of this study.

A total of 62 patients who received neoadjuvant chemotherapy (NAC) were enrolled in this study, with 39 of them failing to achieve pathological complete response (pCR). Inclusion criteria: a). Patients diagnosed with breast cancer and pathologically confirmed TNBC subtype after puncture. b). Patients are standardized to receive neoadjuvant chemotherapy and complete the entire NAC patient. c). Patients with complete pre- and post-treatment ultrasound images of NAC. d). Patients who voluntarily sign the informed consent to participate in the study. Exclusion Criteria: a). Patients who did not complete the entire course of NAC. b). Patients who had previously received chemotherapy or radiotherapy. c). Patients with incomplete or missing medical records, imaging or pathology data. d). Patients with significant comorbidities or other diseases that may affect the efficacy of chemotherapy. e). Patients who refuse to sign the informed consent. Ultrasound images taken before and after neoadjuvant therapy, along with clinical information, were collected from these patients. Transcriptomic (Fragments per kilobase of transcript per million; FPKM) and clinical data of breast cancer patients were obtained from The Cancer Genome Atlas (TCGA; https://www.cancer.gov/ccg/research/genome-sequencing/tcga) (accessed on 25 September 2024). The preprocessing steps included the exclusion of low-expression genes and data normalization. We integrated 1,336 mitochondrial-related gene sets, sourced from existing literature, for subsequent analyses. The validation dataset was obtained from GSE58812 (https://www.ncbi.nlm.nih.gov/geo/query/acc.cgi?acc=GSE58812) (accessed on 25 September 2024), including 107 TNBC samples. The workflow is illustrated in Fig. 1.

### Differential expression gene analysis

Using the profile of 1366 mitochondrial-related genes, we performed differential expression analysis with the edgeR package (version 4.2.1), comparing normal tissue with non-triple-negative breast cancer (no-TNBC), normal tissue with TNBC, and no-TNBC with TNBC. Genes with |log2Fold Change| > 1 and *p* < 0.05 were defined as differentially expressed genes. We then applied the Robust Rank Aggregation (RRA) algorithm to further analyze the differential expression results across the three comparisons, identifying significant differentially expressed mitochondrial genes (DEM) in the three groups.

### Neoadjuvant chemotherapy drug resistance-related ultrasound feature extraction

We conducted feature extraction from ultrasound images using Python, followed by dimensionality reduction analysis using the LASSO regression algorithm to identify key NAC resistance-related ultrasound features. Subsequently, we performed a propensity-matching study to align patient image features with genomic data. DEMs with a correlation greater than 0.1 and *p*-values less than 0.05 were designated as key resistance genes.

### Prognostic model development and evaluation

We defined the outcome event of the model as Overall Survival (OS) and input the identified significant mitochondrial-related genes into a univariate Cox regression model to determine risk factors affecting the prognosis of TNBC patients. These risk factors were then entered into a multivariate Cox regression model to identify independent risk factors influencing patient prognosis. Using the coefficients from the multivariate Cox regression and the corresponding gene expression levels, we constructed a mitochondrial-related gene signature. To verify the model’s robustness, we used the GSE58812 dataset as the test set. Before model validation, we applied the combat package to remove batch effects between the two datasets, thereby eliminating biases. Finally, we evaluated the model’s predictive performance by calculating the area under the receiver operating characteristic curve.

### Prognostic model and clinical variables

To assess the clinical utility of the model, we analyzed the relationship between the gene signature and variables such as age, lymph node status, and tumor staging. We also conducted survival analyses based on different clinical variables to determine survival differences between high- and low-risk patients within subgroups. The log-rank test was used to analyze survival differences between groups.

### Prognostic model and chemotherapy drugs for TNBC

We further examined the sensitivity of high- and low-risk patients in the model to 11 commonly used chemotherapy drugs, aiming to assess the model’s potential in guiding chemotherapy response predictions. The chemotherapy drugs were sourced from the GDSC database, and drug sensitivity was assessed using the R package pRRophetic (version 0.5), with IC_50_ values serving as the sensitivity measure.

### TNBC immunotherapy under strata by prognostic model

Given the importance of immunotherapy as an adjuvant treatment for TNBC, we analyzed immune infiltration in high- and low-risk patients using the ESTIMATE and CIBERSORT algorithms. The distribution of immune checkpoints between the high- and low-risk groups was compared using the Wilcoxon test. Immunotherapy data were obtained from the Imvigor 210 dataset. We first assessed the differences in treatment outcomes between the high- and low-risk groups following immunotherapy. Subsequently, we combined multiple machine learning algorithms to predict immunotherapy responses based on the signature genes.

### Statistical analysis

For continuous variables with a normal distribution, a *t*-test was used to assess differences between groups. For non-normally distributed data, a rank sum test was employed. Survival differences were evaluated using the log-rank test, and univariate and multivariate Cox regression analyses were conducted to identify independent prognostic factors. *p*-values less than 0.05 were considered statistically significant.

## Results

### DEM-related genes in triple-negative breast cancer

**Figure 2:**
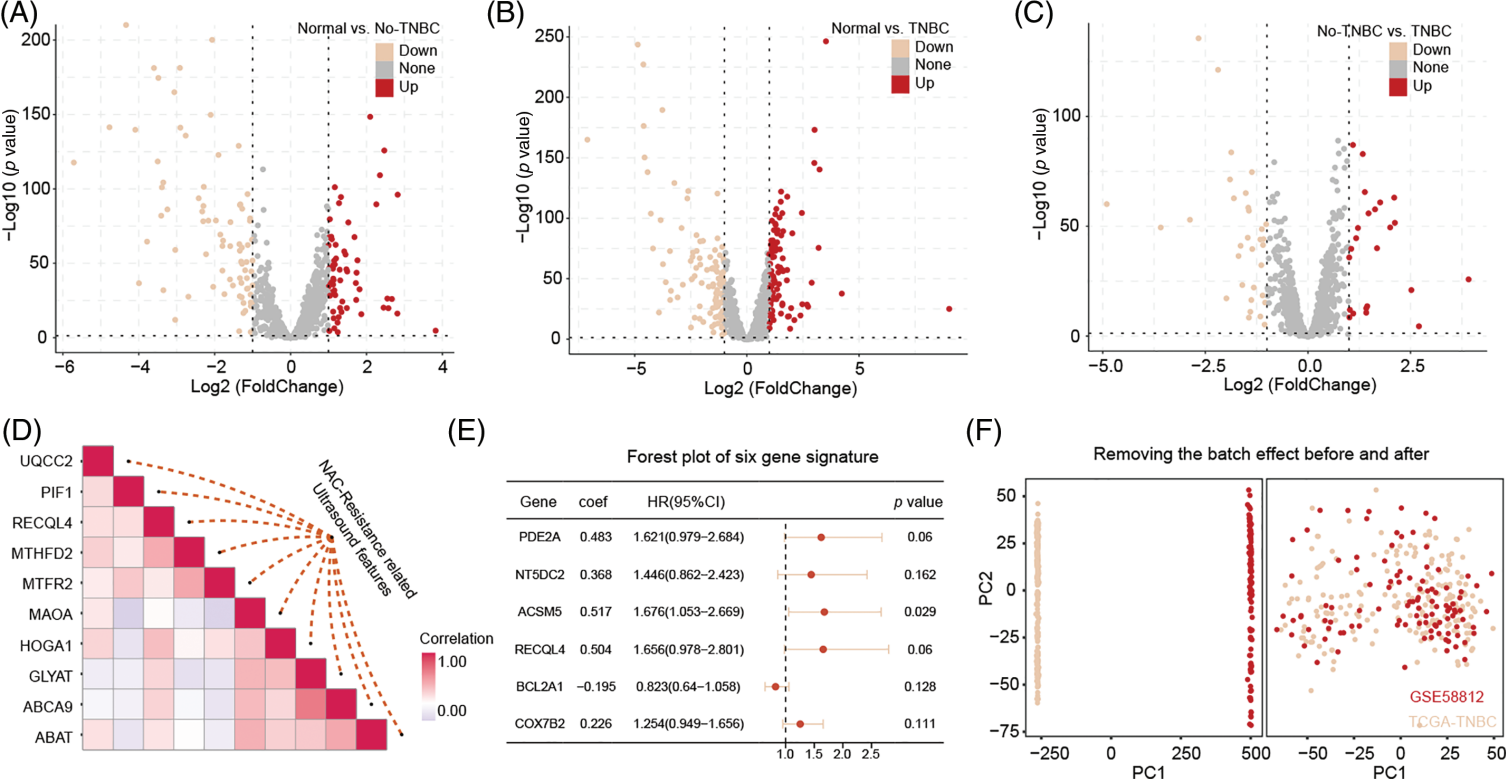
The identification of DEGs related to ultrasound features and the construction of a predictive model. The DEGs between normal *vs*. no-TNBC (A), normal *vs*. no-TNBC (B), and no-TNBC *vs*. no-TNBC (C), and the correction with NAC-resistance related ultrasound features (D). The predicted model was built by above significant correction genes (E). Remove batch effect from TCGA-TNBC and GSE58812 datasets (F).

Differential expression analysis identified 140 genes differentially expressed between normal tissue and non-TNBC tissue, 209 genes between normal tissue and TNBC tissue, and 55 genes between non-TNBC and TNBC tissues (Fig. 2A–C). The RRA algorithm further identified 32 significant genes across these three groups, with the top 10 genes strongly correlated with ultrasound features displayed in Fig. 2D. These significant genes were extracted from the original matrix, forming a new expression matrix for subsequent analysis.

### A six-mitochondrial-related-gene signatures that guides patient stratification

**Figure 3:**
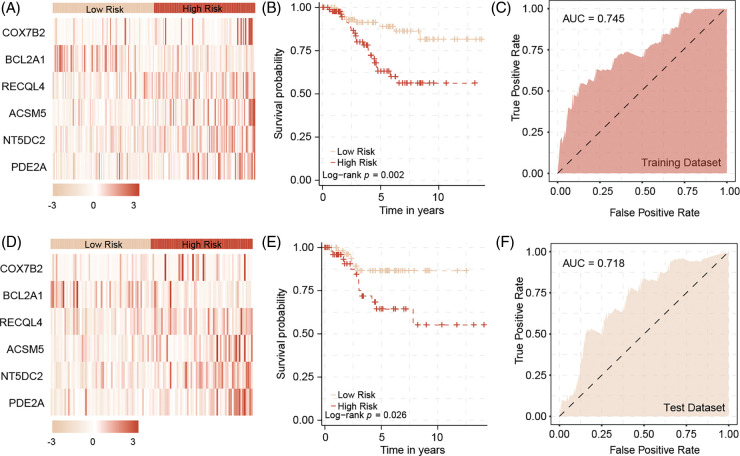
The predictive model in training datasets and test datasets. In the training dataset, the heatmap of six model genes (A), and the survival difference between high and low risk patients (B). The model could predict patients outcome with high accuracy (C). In the test dataset, the heatmap of six model genes (D), and the survival difference between high and low risk patients (E). The model could predict patients outcome with high accuracy by validation (F).

A univariate Cox regression analysis on the new expression matrix revealed that 12 genes are associated with TNBC prognosis. Multivariate Cox regression analysis further identified six genes as independent risk factors affecting TNBC prognosis. Using the regression coefficients and gene expression levels, we constructed a TNBC prognostic model based on these six mitochondrial-related genes (Fig. 2E). To ensure a sufficient sample size, we incorporated the GSE58812 dataset into the study. To prevent data bias, batch effects were removed from both the GSE58812 and the original TCGA datasets (Fig. 2F). Based on the risk scores generated by the model, we categorized patients into high-risk and low-risk groups. Survival analysis of the training set demonstrated that high-risk patients generally have a poorer prognosis compared to low-risk patients, with the model showing high predictive performance (Fig. 3A–C). These findings were further validated in the test set (Fig. 3D–F).

### High-risk patients indicate more advanced tumor characteristics

**Figure 4:**
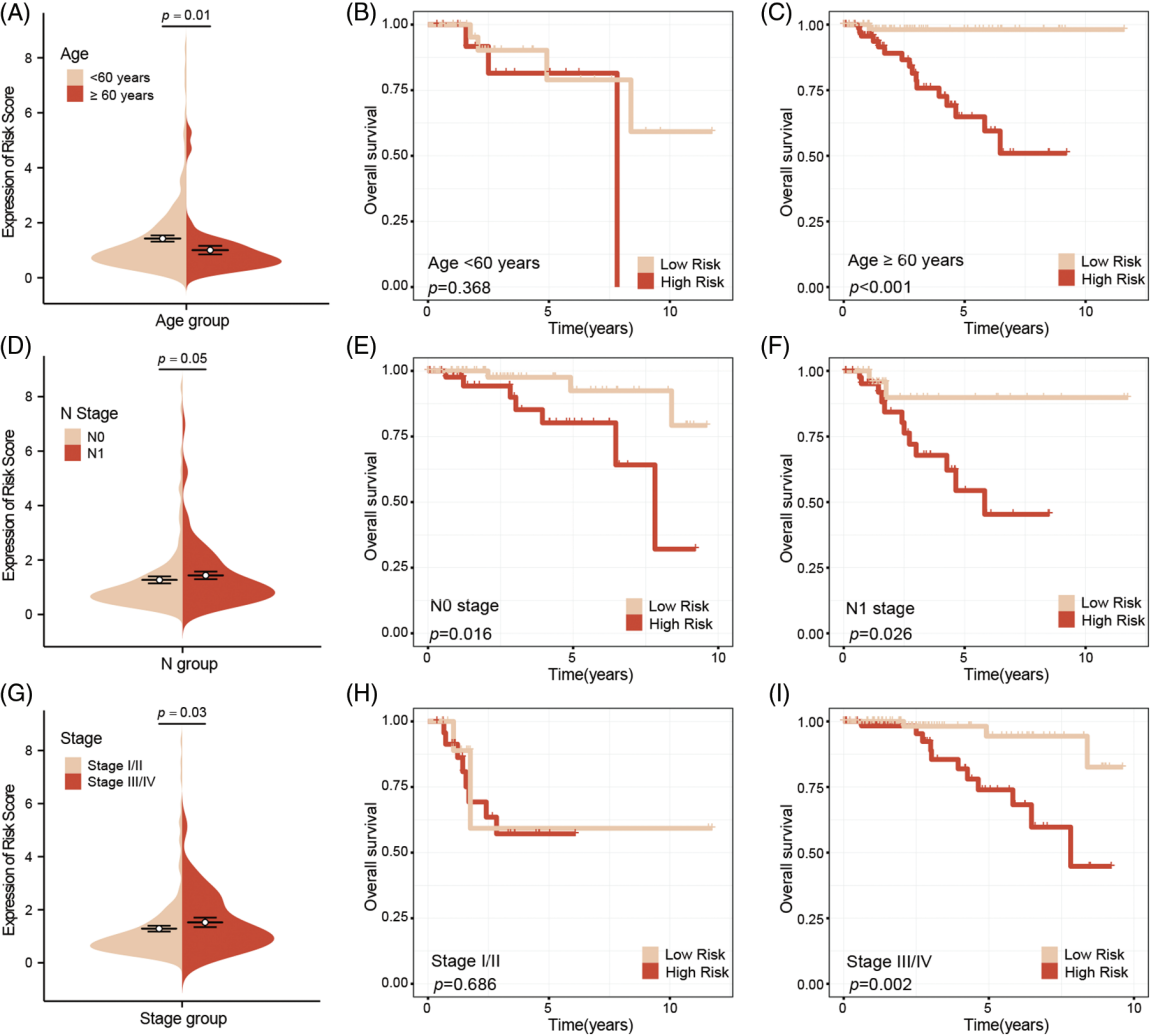
Subgroup survival analysis of clinical factors. Younger patients have higher risk scores compared to older patients (A), and no survival difference between high-risk and low-risk young patients, whereas there was a significant survival difference between two groups of older patients (B, C). High-risk patients are associated with lymph node metastasis, and survival analysis indicated that regardless of lymph node metastasis, high-risk patients have a worse prognosis compared to low-risk patients (D–F). Subgroup analysis based on tumor stage, found that high-risk patients indicate more advanced stages, and poor outcome, this phenomenon was not observation in early-stage breast cancer (G–I).

Subgroup analysis by age suggested that younger patients tend to have higher risk scores compared to older patients (Fig. 4A). However, further analysis revealed no significant survival difference between high-risk and low-risk younger patients, while a significant survival difference was observed between high-risk and low-risk older patients (Fig. 4B–C). Our analysis also confirmed that high-risk patients are more likely to be associated with lymph node metastasis. Subgroup survival analysis indicated that, regardless of lymph node status, high-risk patients consistently have a worse prognosis compared to low-risk patients (Fig. 4D–F). A subgroup analysis based on tumor stage similarly showed that high-risk patients tend to present with more advanced tumor stages. High-risk patients also exhibited poorer prognoses in advanced stages, although this trend was not observed in early-stage breast cancer (Fig. 4G–I).

### Low-risk patients are more sensitive to common chemotherapy drugs

**Figure 5:**
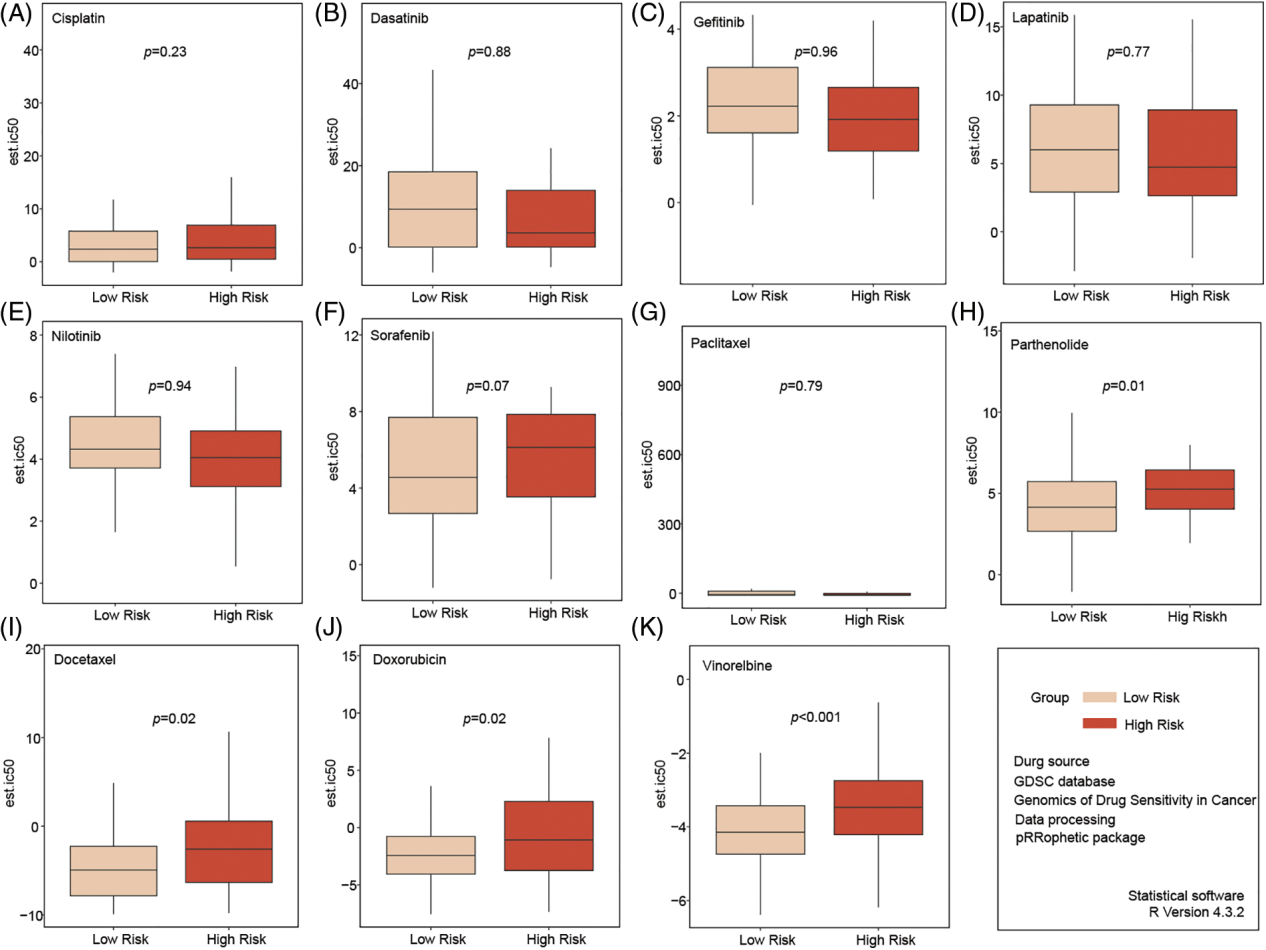
Analysis of chemotherapy drug sensitivity in high- and low-risk groups. No significant in sensitivity to Cisplatin, Dasatinib, Gefitinib, Lapatinib, Nilotinib, Sorafenib, and Paclitaxel between the two groups (A–G). Compared to high-risk patients, low-risk patients seemed more sensitive to Parthenolide, Docetaxel, Doxorubicin, and Vinorelbine (H–K).

We selected 11 commonly used chemotherapy drugs from the GDSC database (https://www.cancerrxgene.org/) (accessed on 25 September 2024) and analyzed the sensitivity of high-risk and low-risk patients with TNBC to these drugs. No differences in sensitivity were observed for Cisplatin, Dasatinib, Gefitinib, Lapatinib, Nilotinib, Sorafenib, and Paclitaxel between the two groups (Fig. 5A–G). However, compared to high-risk patients, low-risk patients appeared to be more sensitive to Parthenolide, Docetaxel, Doxorubicin, and Vinorelbine (Fig. 5H–K).

### Low-risk patients are more sensitive to immunotherapy

**Figure 6:**
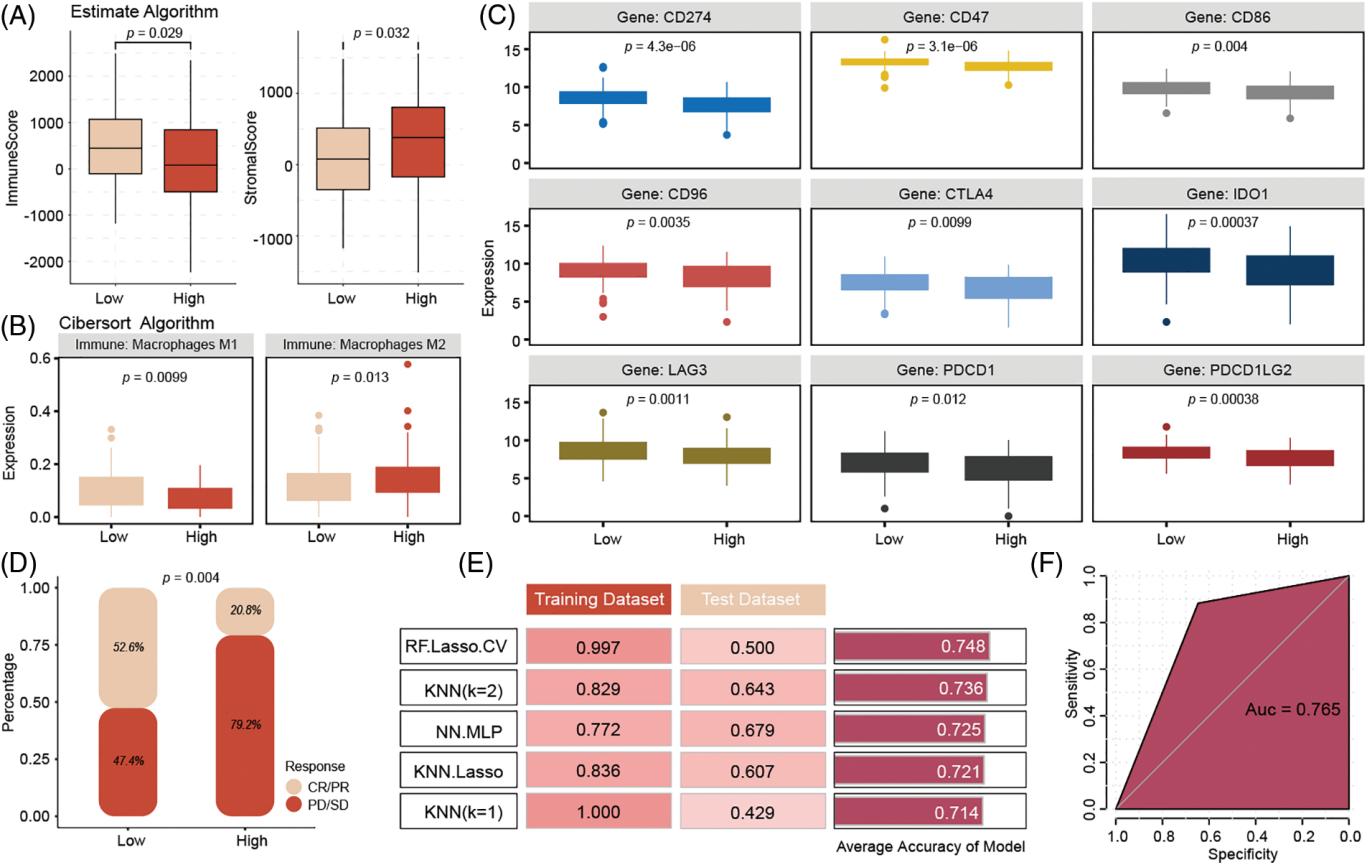
Immune infiltration analysis of high- and low-risk groups in the model and drug sensitivity prediction of model genes. Compared to high-risk patients, the low-risk patient has higher immune infiltration with higher Macrophages M2 infiltration (A, B). Low-risk patients have positive expression with immune checkpoints, (C) and get more CR/PR rates (D) from ICIs therapy. k-nearest neighbors (KNN) model (K = 2) achieved the best predictive performance, with a training set accuracy of 0.829, testing set accuracy of 0.643, to predict the immune therapy response (E, F).

The ESTIMATE algorithm’s immune infiltration analysis indicated that low-risk patients have higher immune infiltration and stromal scores compared to high-risk patients (Fig. 6A). The CIBERSORT algorithm results suggested that high-risk patients exhibit higher infiltration levels of M2 macrophages (Fig. 6B). Analysis of common immune checkpoints revealed that low-risk patients have relatively higher expression of immune checkpoints, suggesting that they may benefit more from immunotherapy (Fig. 6C). Additionally, analysis of the Imvigor 210 database, using the IMvigor210CoreBiologies package (version 2.0.0), showed that low-risk patients who received immunotherapy had higher complete response (CR)/PR rates (Fig. 6D).

### Signature-related genes predict immunotherapy response accuracy

To enhance the clinical application of the model, we input the six genes from the signature into various machine-learning models to identify the best model for accurately predicting immunotherapy response. The results indicated that the k-nearest neighbors model (K = 2) achieved the best predictive performance, with a training set accuracy of 0.829, a test set accuracy of 0.643, and a training set area under the curve (AUC) of 0.765 (Fig. 6E–F).

## Discussion

Despite TNBC’s initial sensitivity to chemotherapy, early CR does not correlate with OS, and relapse rates remain high within 3–5 years. Neoadjuvant anthracycline-based chemotherapy achieves a higher pCR in TNBC compared to non-TNBC subtypes, yet metastatic relapse rates are significantly higher. Immunotherapy, particularly ICIs such as pembrolizumab [13], nivolumab [14], atezolizumab [15], and ipilimumab [16], has shown promise in other cancers and represents a hopeful strategy for TNBC. However, not all patients with TNBC benefit from these new treatment options [17], highlighting the importance of identifying patient subgroups that are most likely to benefit from different therapies.

Previous research has firmly established the link between mitochondrial gene function and breast cancer progression [18]. In TNBC, overexpression of mitochondrial ERβ (mitoERβ) has been demonstrated to suppress tumor proliferation, implying that mitoERβ regulation may be critical in preventing recurrence and developing targeted therapies for TNBC [18]. Our study identified *PDE2A*, *NT5DC2*, *ACSM5*, *RECQL4*, *BCL2A1*, and *COX7B2* as pivotal genes closely linked to OS in TNBC patients. *PDE2A*, a phosphodiesterase responsible for regulating mitochondrial respiration and mitogenic clearance [19], is highly expressed in TNBC but is downregulated following NAC [20]. Notably, another independent TNBC cohort also exhibited *PDE2A* downregulation [19], suggesting that the discrepancy may stem from TNBC’s inherent heterogeneity. *NT5DC2*, encoding the 5’-nucleotidase domain-containing protein 2, plays a role in nucleotide metabolism [21]. Research has demonstrated that *NT5DC2* is upregulated in TNBC, promoting tumor progression and neuropathic pain through interactions with the epidermal growth factor receptor (*EGFR*) to activate downstream signaling. Knockdown of *NT5DC2* in TNBC cells reduces proliferation, migration, invasion, and metabolic activity, while also inhibiting tumor growth and neuropathic pain in mouse models by deactivating the EGFR pathway [19]. *ACSM5*, a member of the Acyl-CoA Synthetase (ACS) enzyme family, was previously identified via TCGA data as a significant breast cancer biomarker, with reduced expression in malignant tissues [22]. In our study, which integrates TCGA and GSE58812 data, *ACSM5* was re-evaluated and confirmed as a risk factor influencing TNBC survival, underscoring the unique biological aspects of TNBC captured by our comprehensive genetic and phenotypic dataset. Furthermore, the 6-gene signature identified in our study effectively stratifies TNBC patients into high- and low-risk groups, with low-risk patients exhibiting significantly better prognoses and demonstrating superior predictive accuracy compared to previously established signatures [23]. Functional annotation of the DEGs between the two risk groups revealed enrichment in metabolic-related biological processes and pathways. Collectively, these studies, along with our findings, underscore the involvement of mitochondrial-related genes in TNBC onset and progression, and their association with disease prognosis.

Recent studies have underscored the pivotal role of mitochondria in promoting tumor chemoresistance, particularly through mechanisms involving dynamin-related protein 1 and high-mobility group box 1 protein [24,25]. While drugs such as atovaquone have demonstrated potential in enhancing chemosensitivity by targeting mitochondrial respiration, the specific relationship between mitochondria and chemoresistance in breast cancer remains insufficiently explored. Our study, in addition to its capacity to predict survival outcomes, identified that a risk score based on a six-gene signature that correlates with the IC_50_ values of traditional chemotherapy drugs, including Docetaxel, Doxorubicin, Parthenolide, and Vinorelbine. Notably, patients classified in the low-risk group exhibited greater sensitivity to these treatments, consistent with their superior OS compared to those in the high-risk group. These findings suggest that our model could be instrumental in identifying TNBC patients likely to benefit from traditional chemotherapy in future clinical practice, representing a significant advancement in personalized cancer treatment.

Immunotherapy has extended survival across various tumor types and offers a promising treatment approach for TNBC. Among the most effective immunotherapeutic strategies are immune ICIs, which target checkpoints such as PD-L1 and PD-1, thereby enhancing the cytotoxicity and proliferation of tumor-infiltrating immune cells [26]. However, translating these findings into clinical practice remains challenging. The phase II KEYNOTE-086 study (NCT02447003) reported an objective response rate of only 5.3% among 170 patients with PD-L1–unselected, previously treated tumors [27], highlighting that a substantial proportion of patients did not benefit from the treatment. This underscores the need to explore the mechanisms influencing immunotherapy efficacy and to identify biological markers that can predict treatment outcomes. Immune metabolism, an emerging concept, refers to the influence of the metabolic state of immune cells on their functional output within the TME, which in turn affects the therapeutic impact of immunotherapy on tumors [28]. A prognostic model based on mitochondrial metabolism-related genes (MMRGs) has established a significant association between 12 MMRGs and breast cancer survival, identifying the MMRG risk score. This model revealed that patients in the low-risk group exhibited higher immune activity and increased sensitivity to specific chemotherapy drugs, suggesting the potential for personalized therapeutic strategies based on MMRG profiles [29]. Unlike the study mentioned, our approach not only employs mitochondrial-related signatures to predict treatment responses but also further investigates immune infiltration and common immune cell checkpoints associated with different risk scores. This provides novel insights into the immunotherapy of TNBC. Our findings indicate that a higher abundance of immune cells and lower levels of M2 macrophages are both linked to a better prognosis, consistent with previous studies [30]. Notably, the low-risk group in our study demonstrated higher expression of immune checkpoints, including *CD274 (PD-L1)*, *CD47*, *CTLA4*, and *PDCD1* (*PD-1*). Despite the immunosuppressive environment induced by immune checkpoints, TNBC has been identified as a key target for immunotherapy [31]. Additionally, research has shown that PD-L1 can serve as a favorable prognostic marker for patients with early-stage TNBC, and its elevated expression in residual disease following NAC suggests a strong potential for using immune checkpoint inhibitors in the post-neoadjuvant treatment setting [32]. To reconcile these seemingly contradictory conclusions, scholars have suggested that while certain immune markers such as PD-1, PD-L1, and OX40L expressed in immune cells correlate with longer progression-free survival (PFS), OX40 expression in tumor cells is associated with shorter PFS [33]. The differential expression of these markers across various cell types, leading to varying prognoses, may explain our results. However, it is evident that due to the high expression of immune checkpoints, patients in the low-risk group are more likely to benefit from ICI treatment. This conclusion has been validated through the model we constructed, demonstrating the potential efficacy of tailored immunotherapy approaches based on immune profiling.

In summary, we developed a model based on mitochondrial-related gene signatures that not only predicts patient prognosis but also forecasts the efficacy of traditional chemotherapy and immunotherapy, thereby aiding in treatment stratification. The model exhibited robust predictive capabilities. However, our study has several limitations. First, TNBC is a highly heterogeneous tumor, and the stability and validity of our model require further validation in larger and more diverse cohorts. Second, given the complexity of the TME, our study’s reliance on bulk RNA data precluded the evaluation of gene expression differences at the single-cell level. This limitation prevents the discrete analysis of immune checkpoint markers in tumor cells *vs*. those in the TME, potentially introducing biases into our findings. Third, although our results suggest a role for mitochondrial-related genes in the progression and treatment response of TNBC, the precise mechanisms underlying these associations require further experimental validation.

## Conclusion

We identified resistance-related features from ultrasound images and integrated them with genomic data, enabling effective risk stratification of patients and prediction of the efficacy of NAC and immunotherapy in TNBC.

## Data Availability

The datasets generated during and/or analyzed during the current study are available from the corresponding author on reasonable request.

## References

[1] Sung H, Ferlay J, Siegel RL, Laversanne M, Soerjomataram I, Jemal A, et al. Global cancer statistics 2020: GLOBOCAN estimates of incidence and mortality worldwide for 36 cancers in 185 countries. CA: A Cancer J Clin. 2021;71(3):209–49. 10.3322/caac.21660 33538338

[2] Nolan E, Lindeman GJ, Visvader JE. Deciphering breast cancer: from biology to the clinic. Cell. 2023;186(8):1708–28. doi:10.1016/j.cell.2023.01.040; 36931265

[3] Wang J, Wu S-G. Breast cancer: an overview of current therapeutic strategies, challenge, and perspectives. Breast Cancer: Targets Ther. 2023;15:721–30. 10.2147/BCTT.S432526 PMC10596062 37881514

[4] Cuthrell KM, Tzenios N. Breast cancer: updated and deep insights. Int Res J Oncol. 2023;6(1):104–18.

[5] Yin L, Duan J-J, Bian X-W, Yu S-C. Triple-negative breast cancer molecular subtyping and treatment progress. Breast Cancer Res. 2020;22(1):1–13. 10.1186/s13058-020-01296-5 PMC7285581 32517735

[6] Liu S, Xu H, Feng Y, Kahlert UD, Du R, Torres-de la Roche LA, et al. Oxidative stress genes define two subtypes of triple-negative breast cancer with prognostic and therapeutic implications. Front Genet. 2023;14:1230911. doi:10.3389/fgene.2023.1230911; 37519893 PMC10372428

[7] Jiang Y-Z, Ma D, Suo C, Shi J, Xue M, Hu X, et al. Genomic and transcriptomic landscape of triple-negative breast cancers: subtypes and treatment strategies. Cancer Cell. 2019;35(3):428–40.e5. doi:10.1016/j.ccell.2019.02.001; 30853353

[8] Yan L-R, Wang A, Lv Z, Yuan Y, Xu Q. Mitochondria-related core genes and TF-miRNA-hub mrDEGs network in breast cancer. Biosci Rep. 2021;41(1):BSR20203481. doi:10.1042/BSR20203481; 33439992 PMC7843495

[9] Li Y, Li Z. Potential mechanism underlying the role of mitochondria in breast cancer drug resistance and its related treatment prospects. Front Oncol. 2021;11:629614. doi:10.3389/fonc.2021.629614; 33816265 PMC8013997

[10] Munkácsy G, Santarpia L, Győrffy B. Therapeutic potential of tumor metabolic reprogramming in triple-negative breast cancer. Int J Mol Sci. 2023;24(8):6945. doi:10.3390/ijms24086945; 37108109 PMC10138520

[11] Shinde A, Jung H, Lee H, Singh K, Roy M, Gohel D, et al. TNF-α differentially modulates subunit levels of respiratory electron transport complexes of ER/PR+ ve/−ve breast cancer cells to regulate mitochondrial complex activity and tumorigenic potential. Cancer Metab. 2021;9(1):19. doi:10.1186/s40170-021-00254-9. 33926547 PMC8082668

[12] Al Haq AT, Tseng H-Y, Chen L-M, Wang C-C, Hsu H-L. Targeting prooxidant MnSOD effect inhibits triple-negative breast cancer (TNBC) progression and M2 macrophage functions under the oncogenic stress. Cell Death Dis. 2022;13(1):49. doi:10.1038/s41419-021-04486-x; 35017469 PMC8752602

[13] Le DT, Diaz LA Jr, Kim TW, Van Cutsem E, Geva R, Jäger D, et al. Pembrolizumab for previously treated, microsatellite instability-high/mismatch repair-deficient advanced colorectal cancer: final analysis of KEYNOTE-164. Eur J Cancer. 2023;186(3):185–95. doi:10.1016/j.ejca.2023.02.016; 37141828

[14] Sanai FM, Odah HO, Alshammari K, Alzanbagi A, Alsubhi M, Tamim H, et al. Nivolumab as second-line therapy improves survival in patients with unresectable hepatocellular carcinoma. Cancers. 2024;16(12):2196. doi:10.3390/cancers16122196; 38927902 PMC11202187

[15] Reddy SM, Carroll E, Nanda R. Atezolizumab for the treatment of breast cancer. Expert Rev Anticancer Ther. 2020;20(3):151–8. doi:10.1080/14737140.2020.1732211; 32067545

[16] Tintelnot J, Stein A, Paschold L, Goekkurt E, Schultheiß C, Thuss-Patience PC, et al. Final survival results of ipilimumab or FOLFOX in combination with nivolumab and trastuzumab in previously untreated HER2 positive esophago gastric adenocarcinoma: the randomized AIO INTEGA trial. Am Soc Clin Oncol. 2023;16:4026–6.

[17] Marra A, Viale G, Curigliano G. Recent advances in triple negative breast cancer: the immunotherapy era. BMC Med. 2019;17:1–9. 31068190 10.1186/s12916-019-1326-5 PMC6507064

[18] Song I-S, Jeong YJ, Jeong SH, Kim JE, Han J, Kim T-H, et al. Modulation of mitochondrial ERβ expression inhibits triple-negative breast cancer tumor progression by activating mitochondrial function. Cell Physiol Biochem. 2019;52(3):468–85. doi:10.33594/000000000. 30873822

[19] Lobo MJ, Reverte-Salisa L, Chao Y-C, Koschinski A, Gesellchen F, Subramaniam G, et al. Phosphodiesterase 2A2 regulates mitochondria clearance through Parkin-dependent mitophagy. Commun Biol. 2020;3(1):596. doi:10.1038/s42003-020-01311-7; 33087821 PMC7578833

[20] Tang X, Thompson KJ, Kalari KR, Sinnwell JP, Suman VJ, Vedell PT, et al. Integration of multi-omics data shows downregulation of mismatch repair, purin, and tublin pathways in AR-negative triple-negative chemotherapy-resistant breast tumors. bioRxiv. 2022. doi:10.1101/2022.05.16.492190. PMC10207800 37226243

[21] Jia Y, Li J, Wu H, Wang W, Sun S, Feng C, et al. Comprehensive analysis of NT5DC family prognostic and immune significance in breast cancer. Medicine. 2023;102(6):e32927. doi:10.1097/MD.0000000000032927; 36820551 PMC9907984

[22] Yazdani B, Jazini M, Jabbari N, Karami M, Rahimirad S, Azadeh M, et al. Altered expression level of ACSM5 in breast cancer: an integrative analysis of tissue biomarkers with diagnostic potential. Gene Rep. 2021;22(1):100992. doi:10.1016/j.genrep.2020.100992.

[23] Wang Y, Wang D-Y, Bu K-N, Gao J-D, Zhang B-L. Prognosis prediction and risk stratification of breast cancer patients based on a mitochondria-related gene signature. Sci Rep. 2024;14(1):2859. doi:10.1038/s41598-024-52981-w; 38310106 PMC10838276

[24] Guerra F, Arbini AA, Moro L. Mitochondria and cancer chemoresistance. Biochimica et Biophysica Acta (BBA)-Bioenergetics. 2017;1858(8):686–99. doi:10.1016/j.bbabio.2017.01.012; 28161329

[25] Hosseini M, Rezvani HR, Aroua N, Bosc C, Farge T, Saland E, et al. Targeting myeloperoxidase disrupts mitochondrial redox balance and overcomes cytarabine resistance in human acute myeloid LeukemiaMPO in chemoresistance. Cancer Res. 2019;79(20):5191–203. doi:10.1158/0008-5472.CAN-19-0515; 31358527

[26] Dermani FK, Samadi P, Rahmani G, Kohlan AK, Najafi R. PD-1/PD-L1 immune checkpoint: potential target for cancer therapy. J Cell Physiol. 2019;234(2):1313–25. doi:10.1002/jcp.27172; 30191996

[27] Adams S, Schmid P, Rugo H, Winer E, Loirat D, Awada A, et al. Pembrolizumab monotherapy for previously treated metastatic triple-negative breast cancer: cohort A of the phase II KEYNOTE-086 study. Ann Oncol. 2019;30(3):397–404. doi:10.1093/annonc/mdy517; 30475950

[28] Leone RD, Powell JD. Metabolism of immune cells in cancer. Nat Rev Cancer. 2020;20(9):516–31. doi:10.1038/s41568-020-0273-y; 32632251 PMC8041116

[29] Lin Y, Huang Z, Zhang B, Yang H, Yang S. Construction and analysis of a mitochondrial metabolism-related prognostic model for breast cancer to evaluate survival and immunotherapy. J Membr Biol. 2024;257(1):63–78. doi:10.1007/s00232-024-00308-1; 38441572

[30] Yang A, Wu M, Ni M, Zhang L, Li M, Wei P, et al. A risk scoring system based on tumor microenvironment cells to predict prognosis and immune activity in triple-negative breast cancer. Breast Cancer. 2022;29(3):468–77. doi:10.1007/s12282-021-01326-w; 35061208 PMC9021102

[31] Tarekegn K, Keskinkilic M, Kristoff TJ, Evans ST, Kalinsky K. The role of immune checkpoint inhibition in triple negative breast cancer. Expert Rev Anticancer Ther. 2023;23(10):1095–106. doi:10.1080/14737140.2023.2265059; 37771270

[32] Dieci MV, Tsvetkova V, Griguolo G, Miglietta F, Tasca G, Giorgi CA, et al. Integration of tumour infiltrating lymphocytes, programmed cell-death ligand-1, CD8 and FOXP3 in prognostic models for triple-negative breast cancer: analysis of 244 stage I-III patients treated with standard therapy. Eur J Cancer. 2020;136:7–15. doi:10.1016/j.ejca.2020.05.014; 32622323

[33] Choi JE, Lee JS, Jin M-S, Nikas IP, Kim K, Yang S, et al. The prognostic value of a combined immune score in tumor and immune cells assessed by immunohistochemistry in triple-negative breast cancer. Breast Cancer Res. 2023;25(1):134. doi:10.1186/s13058-023-01710-8; 37924153 PMC10625207

